# COVID-19 in a pediatric cohort—retrospective review of chest computer tomography findings

**DOI:** 10.1186/s43055-021-00461-w

**Published:** 2021-03-24

**Authors:** Rita Pina Prata, Ana Forjaco, Carina A. Ruano, João Lopes Dias, Lúcia Fernandes, Alexandra Ferreira, Pedro Alves, Rita Cabrita Carneiro, Ana Nunes, Eugénia Soares

**Affiliations:** 1 Radiology Department, Hospital Dona Estefânia, Centro Hospitalar Universitário de Lisboa Central, R. Jacinta Marto 8A, 1169-045 Lisbon, Portugal; 2 Radiology Department, Hospital de Santa Marta, Centro Hospitalar Universitário de Lisboa Central, Lisbon, Portugal; 3 Radiology Department, Hospital de São José, Centro Hospitalar Universitário de Lisboa Central, Lisbon, Portugal

**Keywords:** Coronavirus, Pediatrics, Diagnostic imaging

## Abstract

**Background:**

Radiological features of the novel 2019 coronavirus disease (COVID-19) have been mainly described in adults. Available literature states that imaging findings in children are similar but less pronounced. The aim of this study is to describe and illustrate the chest computer tomography (CT) features of pediatric COVID-19.

**Results:**

This retrospective study was based on the review of all the chest CTs performed in pediatric patients with confirmed COVID-19 disease between March 8th and May 26th 2020 (*n* = 24). The presence of comorbidities and coinfection was assessed, as well as timing of CT examination in relation to the onset of symptoms. CT findings were categorized as typical, indeterminate, atypical, and negative for COVID-19 according to International Expert Consensus Statement on Chest Imaging in Pediatric COVID-19 Patient Management. This study found that CT findings were abnormal in 17 (71%) patients, with 5 (21%), 9 (38%), and 3 (13%) patients considered to have typical, indeterminate, and atypical findings, respectively. The most common CT patterns were multiple ground-glass opacities (58%), followed by consolidations (50%). Six patients showed predominantly peripheral distribution of parenchymal abnormalities. A halo sign was identified in 3 patients and a perilobular pattern was identified in one of the cases with typical findings.

**Conclusions:**

Chest CT findings in children infected with SARS-CoV-2 can be subtle or absent. Besides recognizing typical findings, radiologists should be able to identify features that favor different or concomitant diagnosis.

## Background

After being first isolated in December 2019 in China, the novel coronavirus (SARS-CoV-2) infection has rapidly expanded globally, creating uncertainties in the management of the COVID-19 viral disease. Consequently, in the past few months, several papers have been published aiming to assess the typical clinical, laboratorial, epidemiological, and radiological features of this disease. Typical clinical presentation mirrors other viral pneumonias and includes fever, fatigue, cough, runny nose, dyspnea, and on chest radiographs, pulmonary parenchymal opacities [1]. Increasing data suggest that patients get worse during the second week of symptoms [2], when the immune response of the host is exacerbated [3]. Indeed, following an initial week of mild upper respiratory symptoms (“viremia phase” [3]), lung disease worsens clinically (hypoxemia and dyspnea [2]) and radiologically, peaking at day 10 (ranging 6–11) of illness [4, 5] (“acute (pneumonia) phase” [3]). The predominant pattern on computer tomography (CT) is multifocal ground-glass opacities (GGO), which is typical of lung injury of viral pneumonias [5, 6]. In COVID-19, these are mainly subpleural and predominate in the lower lobes [1, 4]. Consolidation is also seen; however, consolidation without GGO is not typical for COVID-19 [7].

Children of all ages are as likely as adults to get infected [8]. The few studies that focus on pediatric COVID-19 disease demonstrate that children tend to have milder clinical symptoms as well as fewer chest radiographic (CXR) and CT findings, with limited extent of disease in the lungs [1, 9]. Reduced maturity and function of angiotensin-converting enzyme II receptors in children, which are presumed to act as the receptors for the virus to enter the cells, has been proposed as a possible explanation of this milder clinical course [10]. Since in most pediatric patients the clinical presentation of COVID-19 disease is mild, balance between the risk of radiation and necessity for imaging should be evaluated [9]. Actually, imaging is only indicated for patients with moderate to severe acute respiratory illness symptoms or risk factors [8, 11, 12]. Also, CXR has been proven insensitive in mild or early COVID-19 disease [13]. Concerning CT findings in children, published data suggest that, in comparison with adults, pediatric imaging findings are atypical, with less extensive GGO and lower GGO attenuation, with interlobular septal thickening being relatively rare [9]. Bronchial wall thickening and peribronchial distribution of the opacities are more common in children than in adults [1]. Also, in one of the largest pediatric series published (20 patients), consolidation with surrounding ground-glass (halo sign) was common in pediatric patients, which also differs from adults [14].

The scarce literature on appropriateness criteria for imaging and for consensus on reporting CXR and CT findings in children has been challenging in defining pathways and management of patients with suspected COVID-19 disease in Pediatric Centres. While the Fleishner Society Consensus states that children merit separate consideration, particularly with regard to the use of radiation-associated procedures, further recommendations for this age group are scarce [11, 12]. The International Expert Consensus Statement on Chest Imaging in Pediatric COVID-19 Patient Management [8] (herein cited as “International Consensus”) clarified clinical severity, risk factors, and imaging recommendations in pediatric patients with COVID-19. It also described and classified imaging findings as typical, atypical, indeterminate, or negative for COVID-19 disease, suggesting a reporting template.

This study performed on a pediatric cohort with COVID-19 disease, as confirmed with nasopharyngeal swabs positive for SARS-CoV-2 RNA, who had underwent chest CT aims to further contribute to the knowledge of possible imaging abnormalities of this disease.

## Methods

Between March 8th and May 26th 2020, a total of 113 patients tested positive for SARS-CoV-2 and 68 were admitted for in-hospital stay in our tertiary referral pediatric hospital. CT was requested in 24 of these cases based on severe disease criteria as assessed by infectious pediatricians. Only patients with laboratory-confirmed SARS-CoV-2 who underwent CT (Siemens Healthineers Somatom go.Now CT) were included in this retrospective study and none of the cases were excluded.

Age, gender, presenting symptoms (fever, cough, shortness of breath, and others), epidemiological linkage with other COVID-19 patients, presence of comorbidities, and coinfections were evaluated. Timing of the CT examination was correlated with the time course of symptoms.

CT imaging findings were classified as typical, indeterminate, atypical, and normal/negative for COVID-19 according to International Consensus [8] and evaluated accordingly to the following criteria: (1) distribution: unilateral or bilateral opacities, upper or lower predominance (or both), peripheral or central predominance (or both), presence of peribronchial opacities; (2) CT pattern: ground-glass opacities (GGO), crazy paving, consolidation, halo sign (consolidation with a surrounding ground-glass opacity), perilobular opacities and reversed halo sign (both suggestive of secondary organizing pneumonia); (3) other findings: pleural effusion, bronchial wall thickening, tree in bud pattern, centrilobular nodules, and mosaic pattern (suggestive of small airway disease). These CT findings were discussed in our pediatric radiology department weekly meeting and, for this study in particular, were systematically reviewed by a 3rd year radiologist in training and validated by 2 radiologists with more than 20 years of experience in pediatric radiology and a radiologist with 5 years of experience in thoracic radiology.

## Results

### Demographic and clinical data

This cohort of 24 patients had a balanced gender distribution (50% were males) and a mean age of 5.7 years old (range 1 month–18 years; Table 1). The majority of patients (54%) were 2 years old or younger. Seventeen patients (71%) had an epidemiological linkage to other patients with COVID-19. Fourteen patients (58%) had previously known comorbidities, being ex-prematurity (5 patients), and asthma or atopy (4 patients) the most common.

**Table 1:** Demographic and clinical characteristics

	Frequency	%
**Gender**		
Female	12	50%
Male	12	50%
Mean 5,7 years; Median 1 year; (range 1month - 18years)		
Age		
≤ 2 years old	13	54%
3–9 years old	2	8%
10–18 years old	9	38%
**Symptoms** (Five (21%) patients were admitted due to other causes and SARS-CoV-2 was later isolated during in-hospital stay.)		
Fever	16	66%
Cough	13	54%
Shortness of breath	10	42%
Loss of appetite	3	13%
Nasal obstruction, headache, chest pain, odynophagia, rhinorrhea, myalgia	2 each	8%
**Epidemiological linkage with other COVID-19**	17	71%
**Coinfection**	10	42%
**Timing of CT in relation to the onset of symptoms**	Mean 8 days (range 2–22 days)	

Regarding presenting clinical features, 16 (66%) patients had fever, 13 (54%) presented with cough, and 10 (42%) with shortness of breath (Table 1). Five patients were admitted due to other causes and SARS-CoV-2 was later isolated during in-hospital stay. Ten (42%) patients also tested positive for other pathogens besides SARS-CoV-2 (rhinovirus and respiratory syncytial virus (RSV) were the most common).

Patients underwent CT following a median of 8 days after the onset of symptoms (range 2-22 days, *N* = 22). In 2 of the 5 patients that were admitted for other causes besides COVID-19, correlation of the CT timing with time course of symptoms was difficult to assess.

### Imaging findings

CT findings (Table 2) were abnormal in 17 (71%) patients, with 15 presenting bilateral opacities. Concerning distribution of the opacities (Table 2), 25% (6) patients had predominant peripheral distribution. Only 3 patients showed peribronchial opacities. Five (21%) patients had predominantly lower lobe opacities. The most common CT findings were multiple ground-glass opacities which were seen in 14 (58%) patients. In 2 patients, consolidation was the main pattern showing no significant GGO and were classified as atypical. In 3 of the 9 patients that had concurrent GGO and consolidations, consolidation was the predominant pattern. No crazy paving pattern was noted in this series. Three patients had bronchial wall thickening and 4 presented mosaic pattern. Two patients had pleural effusions and one of them had solid pulmonary nodules and mediastinal adenopathies. These findings were classified as atypical, suggesting a different or concomitant diagnosis.

**Table 2:** CT features of COVID-19 patients

			% Total CTs *N* = 24	% Abnormal CTs *N* = 17
Normal/negative CT		7	29%	
Uni/bilateral opacities	Bilateral	15	63%	88%
	Unilateral	2	8%	12%
Lower/upper lobe predominance	Lower	5	21%	29%
	Both	12	50%	71%
Peripheral/central/both	Peripheral	6	25%	35%
	Both	10	42%	59%
Peribronchovascular		3	13%	18%
Bronchial wall thickening		3	13%	18%
Mosaic pattern		4	17%	24%
GGO	0–2 years—8 3–9 years—2 10–18 years—4	14	58%	82%
Halo sign	0–2 years	3	13%	18%
Consolidation	0–2 years—8 3–9 years—2 10–18 years—2	12	50%	71%
GGO + consolidation	0–2 years—6 3–9 years—2 10–18 years—1	9	38%	53%

*CT* computer tomography, *GGO* ground-glass opacities

### CT patterns according to the international consensus [8]

Amongst the 17 patients with abnormal CT (71%), 5 (21%) were considered to have typical findings, 9 (38%) indeterminate, and 3 (13%) atypical features (Table 3). Most of the typical features, including the halo signs observed in this cohort, were seen in patients aged 2 or younger (3/5 patients, Table 3). The reversed halo sign was also observed in a patient of this age group. Typical findings were more frequently observed during the second week of symptoms (3/5 patients) whereas indeterminate features were more commonly seen during the first week of symptoms (5/7 patients, Table 4).

**Table 3:** CT patterns according to the international consensus and age distribution (*N* = 24)

	Normal/negative	Typical	Indeterminate	Atypical
**0–2 years**	4	3	5	1
**3–9 years**	0	1	1	0
**10–18 years**	3	1	3	2
**Total**	**7**	**5**	**9**	**3**
**% total**	**29%**	**21%**	**38%**	**13%**

**Table 4:** CT patterns according to the timing of the CT examination in relation to the onset of symptoms (*N* = 22)

	Normal/negative	Typical	Indeterminate	Atypical	Total
**2–6 days**	4	1	5	1	11
**7–14 days**	3	3	1	2	9
**15–22 days**	0	1	1	0	2
**Total**	7	5	7	3	

#### Typical pattern

**Fig. 1:**
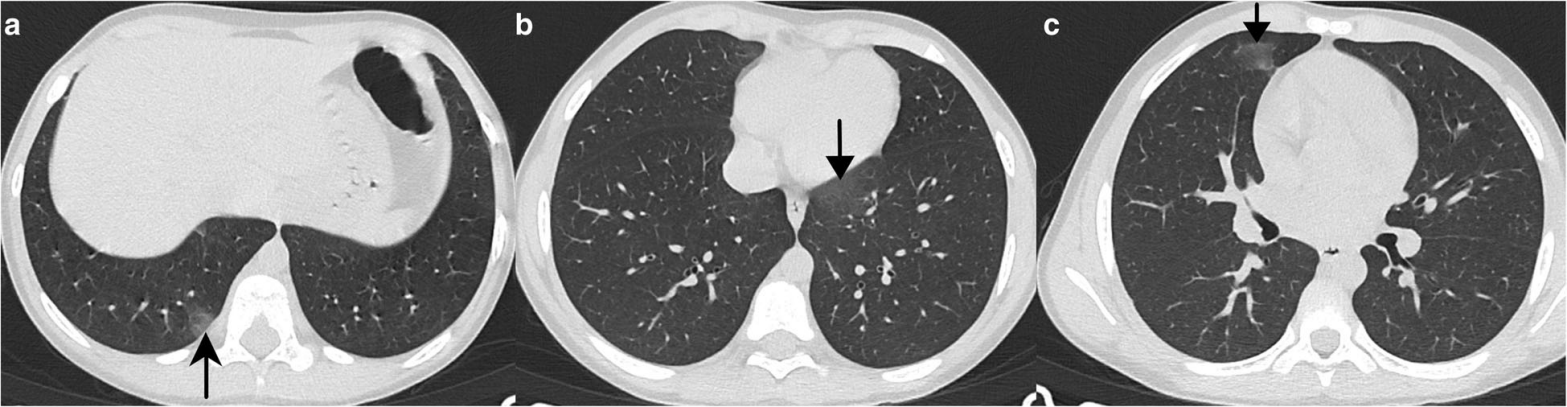
**a**–**c** Typical findings—subpleural ground-glass opacities (arrows), with lower lobe predominance in a 10-year-old asthmatic boy

**Fig. 2:**
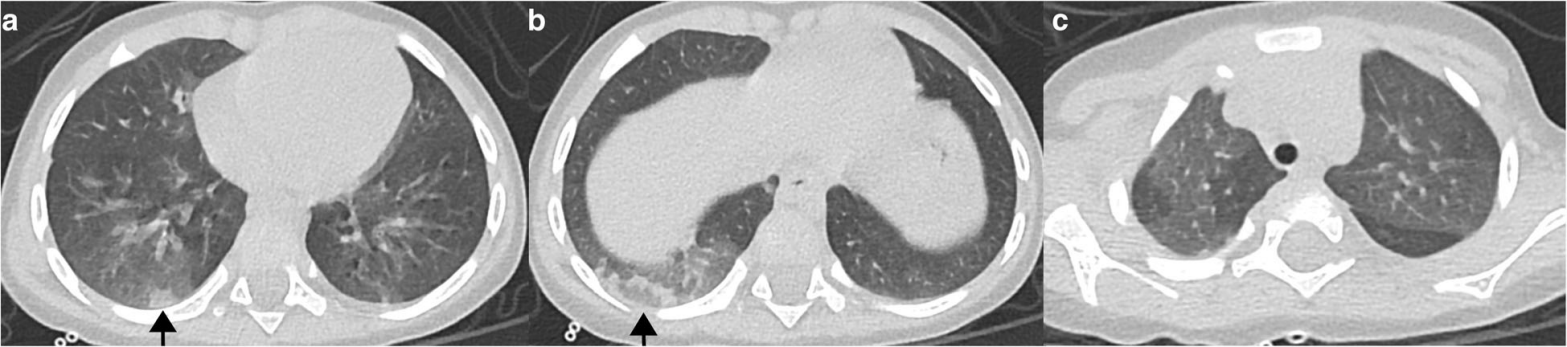
Typical findings in a 1-year-old ex-premature girl. CT shows small subpleural consolidation with surrounding ground-glass opacity (halo sign—arrow in **a**), perilobular peripheral opacity (arrow in **b**), suggestive of secondary organizing pneumonia, with underlying ground-glass opacities. Mosaic attenuation pattern was also seen (**c**) suggesting concomitant small airways disease (probably due to coinfection with other viral diseases, in this case, metapneumovirus)

**Fig. 3:**
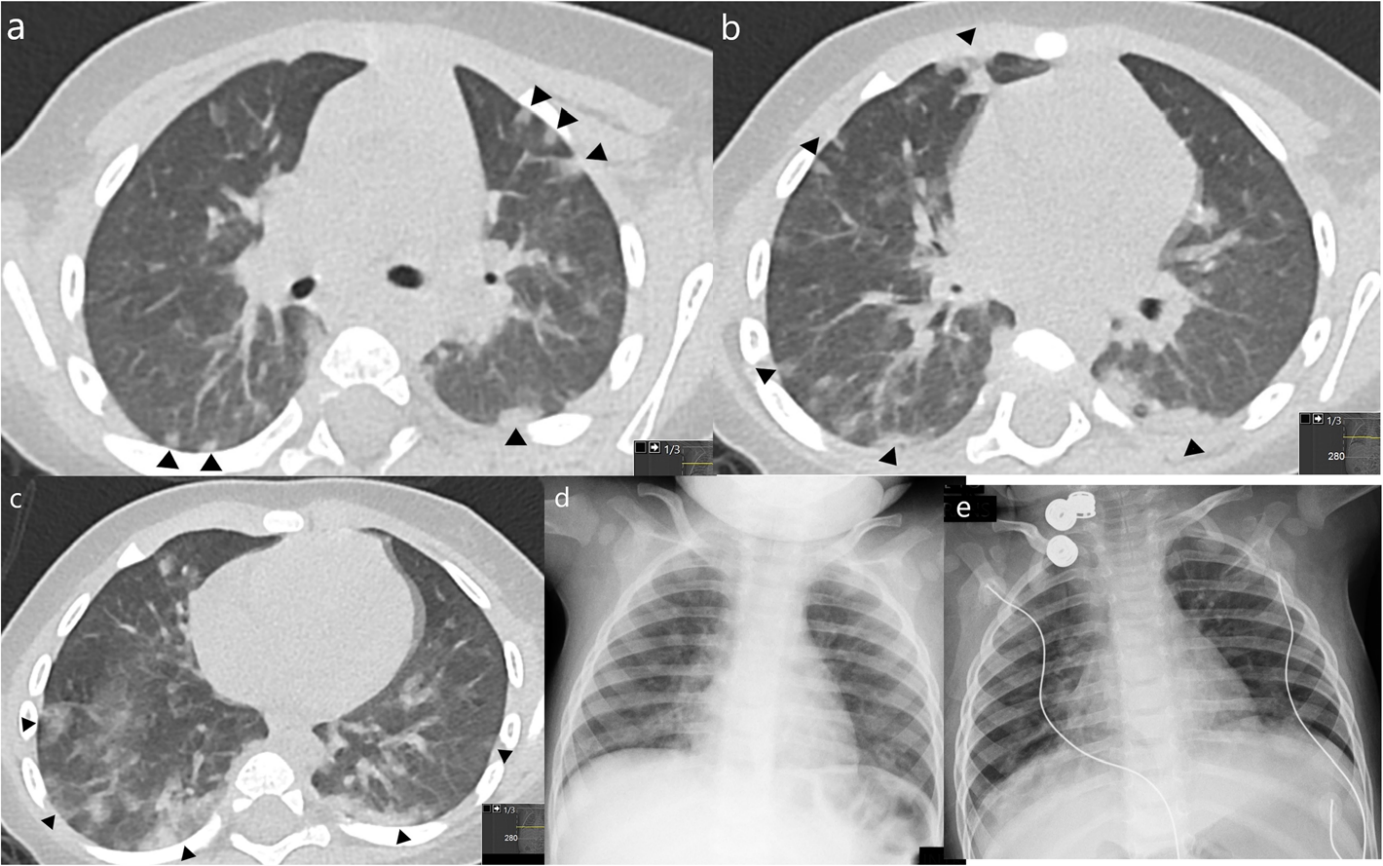
Typical findings in a 10-month-old infant. Patchy consolidations and GGO in the upper and lower lobes predominantly seen in the subpleural pulmonary parenchyma (**a**–**c**). Some of these opacities corresponded to an halo sign. Chest X-ray performed the day before the CT (**d**) and 4 days after (**e**) shows partial resolution of the subtle bilateral opacities, typical for COVID-19

**Fig. 4:**
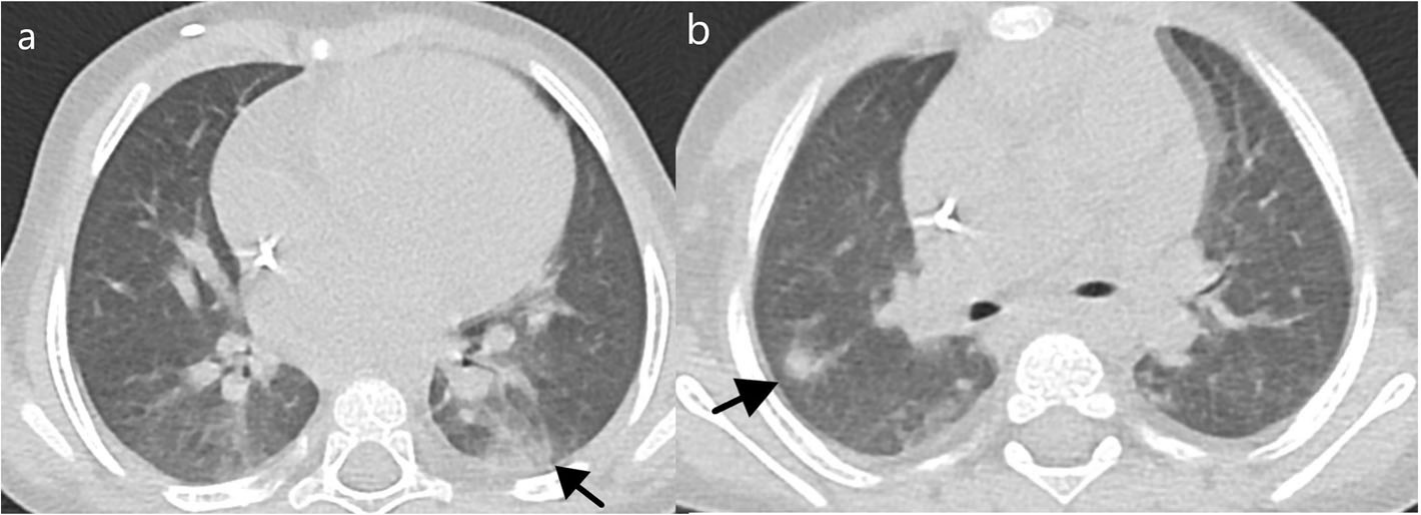
**a**, **b** Typical findings in a 2-year-old ex-premature male with small bowel syndrome—peripheral focal consolidations in the lower and upper lobes. Consolidation was the predominant pattern in this patient

Typical CT findings include bilateral, peripheral GGO, and/or consolidation with lower lobe predominance and “halo sign” [8]. Of the 5 cases with typical CT findings, all had bilateral (one predominant on the right) and peripheral opacities. In these patients, all the opacities predominated in the lower lobes (Figs. 1 and 2), and 3 had both upper and lower lobe opacities (Figs. 3 and 4). One of the patients only presented GGO (Fig. 1), while the others also had consolidations (Figs. 2, 3, and 4). Halo sign was seen in 3 patients and reversed halo sign in one patient (Fig. 2).

#### Indeterminate pattern

**Fig. 5:**
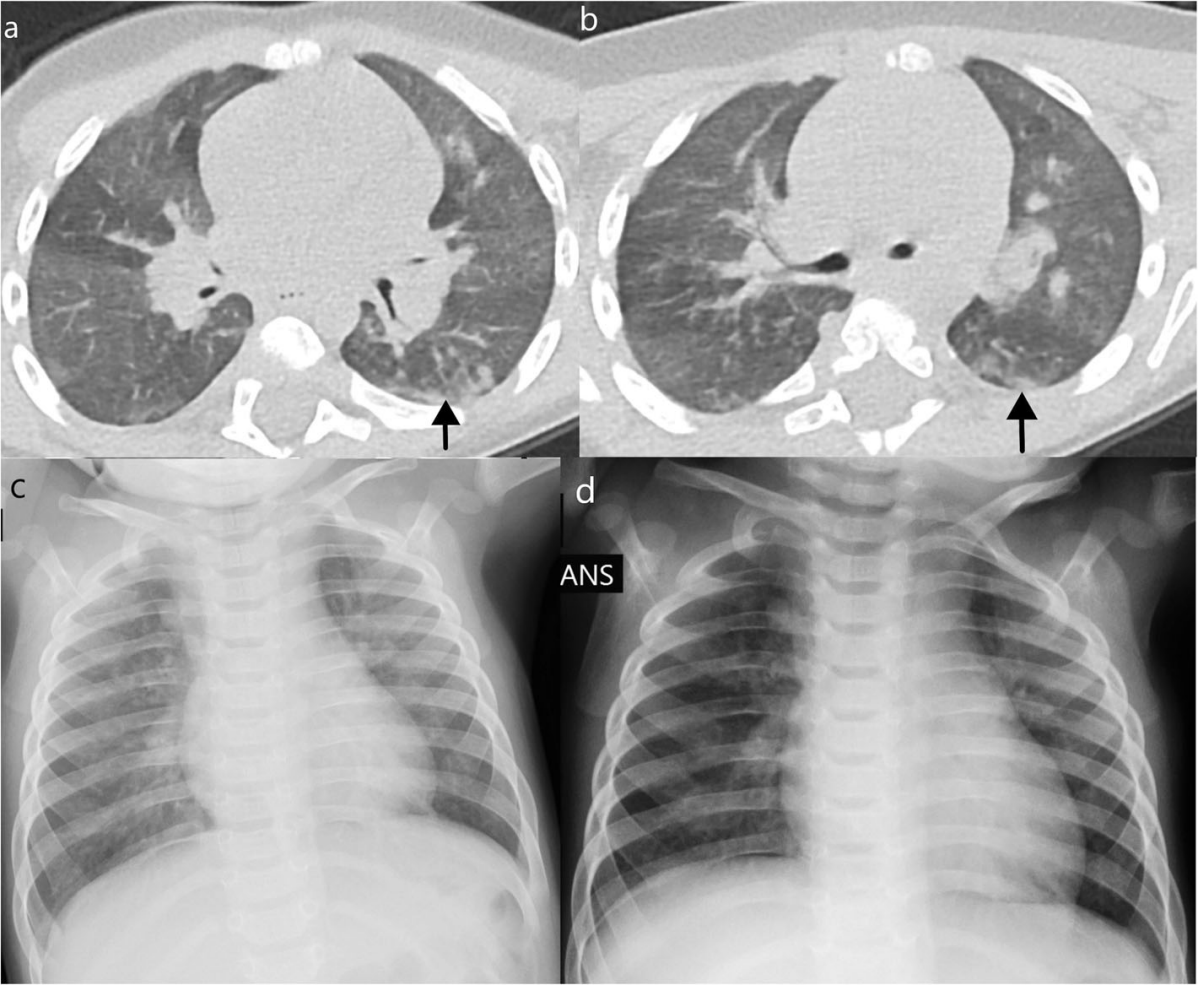
Indeterminate features in a 7-month-old infant. Chest CT on day 5 of symptoms (**a**, **b**) showed diffuse GGOs with no peripheral or central predominance. Bronchial wall thickening was also noted (not shown). This patient was coinfected with adenovirus and RSV which could partially be responsible for the mosaic attenuation pattern/small airway disease. Linear opacities in the posterior subpleural lung (arrows) are commonly seen in infants representing athelectasic bands due to hypoventilation of the decubitus. Chest X-ray from the same patient (**c**) shows diffuse reduced lung transparency bilaterally, with no predominant distribution. Opacities almost completely resolved 8 days later (**d**)

**Fig. 6:**
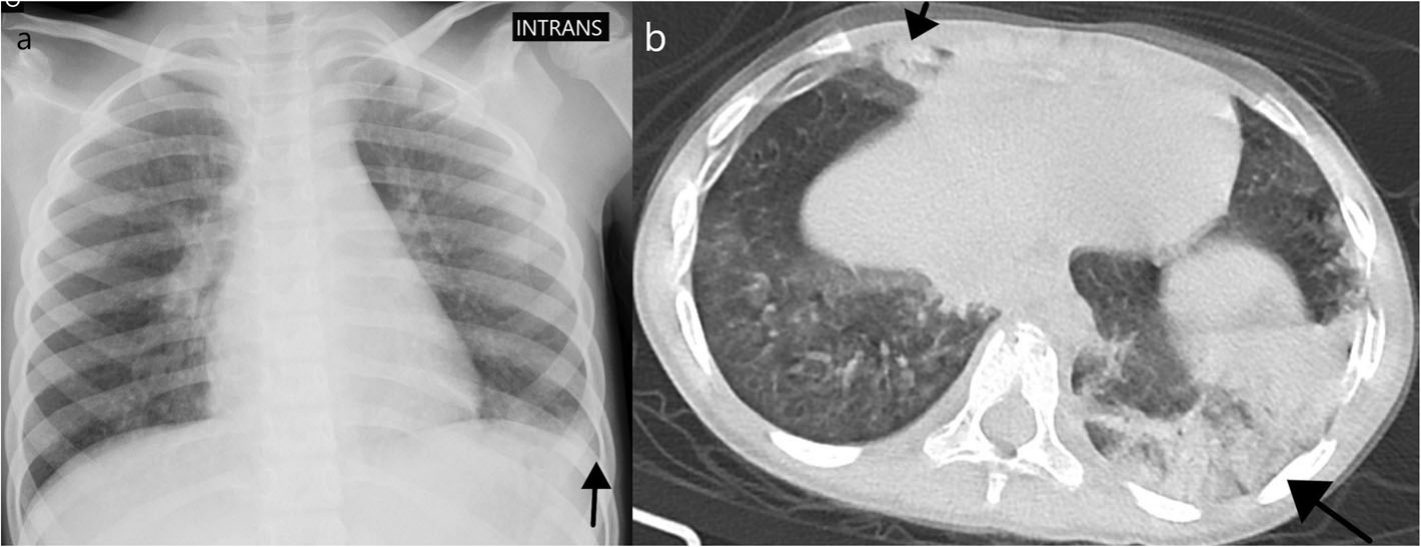
Indeterminate features in a 5-year-old asthmatic girl—CXR showed unilateral lower opacity (**a**), which is not typical for COVID-19. CT (**b**) was performed 6 days after the onset of symptoms, which showed bilateral peripheral consolidations, mainly in the left lower lobe but also in the middle lobe (arrows) and internal segment of the lower right lobe. The predominant pattern is consolidation, which is not typical for COVID-19, although a characteristic peripheral and lower distribution is seen

**Fig. 7:**
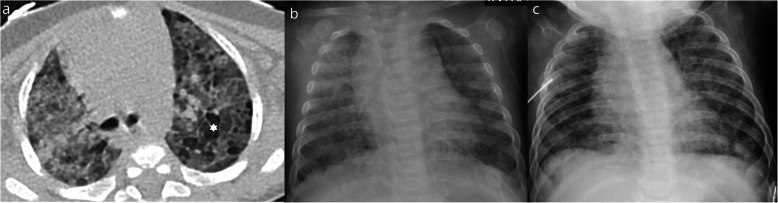
Indeterminate features. Chest CT of a 4-month-old infant on the third day of symptoms (**a**) shows diffuse cystic lucencies throughout both lungs (*), compatible with known bronchopulmonary dysplasia. CT also shows superimposed diffuse ground-glass opacities which could be attributable to the confirmed COVID-19. CXR on the same day as CT (**b**) and 6 days later (**c**) shows improvement of the diffuse ground-glass opacities

**Fig. 8:**
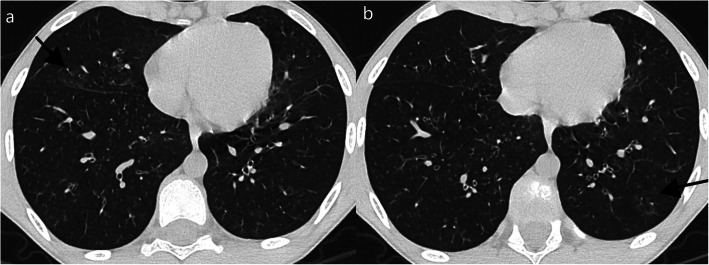
Indeterminate features in a 11-year-old with history of atopy that was coinfected with *Mycoplasma pneumoniae* and rhinovirus. Chest CT shows 2 very subtle nodular GGO, one not peripheral (**a**). Subtle centrilobular micronodules were seen in the lower lobes (**b**) suggesting small airway disease. These findings are not typical for COVID-19 disease. Diffuse bronchial wall thickening was also seen

Indeterminate patterns should raise suspicion for other diagnosis like atypical bacterial and viral pneumonias. Of our 9 indeterminate cases, all except one had bilateral opacities, 7 had both central and peripheral opacities (Fig. 5), 1 had several subtle millimetric peribronchovascular opacities, and 1 had predominant unilateral consolidation (Fig. 6). Consolidation was present in 4 of our 9 indeterminate cases (Fig. 6). One patient presented ground-glass opacities superimposed on underlying diffuse lung disease (Fig. 7), and one patient (Fig. 8) demonstrates very subtle GGO nodules, bronchial wall thickening, and subtle centrilobular micronodules suggesting small airway disease.

#### Atypical pattern

**Fig. 9:**
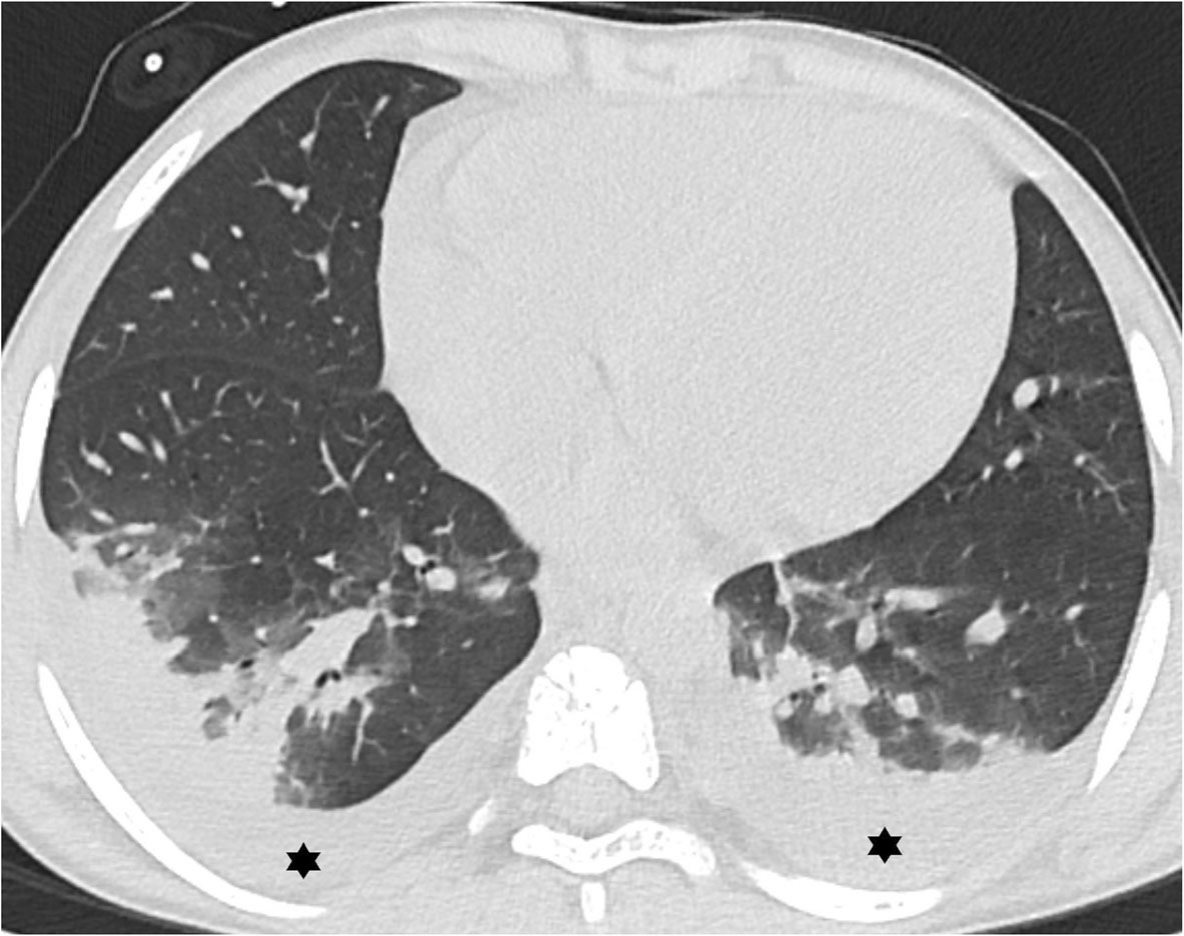
Atypical features in a patient with sickle cell anemia and rt-PCR positivity for COVID-19—bilateral lung effusion (*) and peripheral and dependent consolidation in the lower lobes with scarce ground-glass opacities. This patient also had an increased diameter of the pulmonary artery, pericardial effusion, and mild cardiomegaly (not shown) which could also justify the bilateral effusion due to cardiac insufficiency

**Fig. 10:**
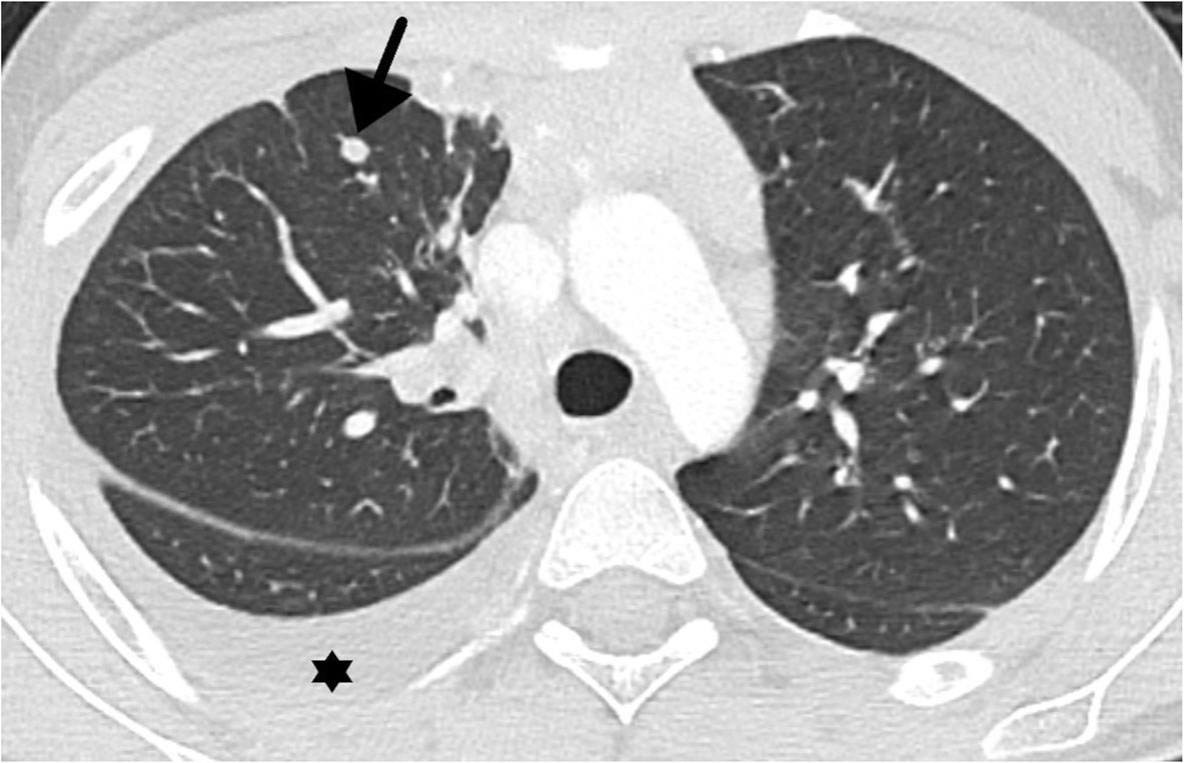
Atypical features in a 11-year-old girl with rt-PCR positivity for SARS-CoV-2 admitted due to polyarthritis and fever—unilateral lung effusion (*) and 2 solid nodules (arrow) as well as mediastinal and hilar adenopathies were documented. CT findings were thought to be associated with rheumatological disease

There were 3 atypical cases in our case series in which an alternative explanation for the CT findings was considered. One had homozygous sickle cell anemia and was admitted due to a vasoocclusive crisis in the elbow and subsequently presumed to have an acute thoracic syndrome (Fig. 9). Another patient with polyarthrirtis for the past month presented with fever and exhibited unilateral pleural effusion, parenchymal nodules, and adenopathies (Fig. 10). The third had bilateral posterior upper and lower lobe consolidations, with no GGO and had multiple comorbidities including ex-prematurity, arterial hypertension, and chronic renal disease.

#### Normal/negative CT

**Fig. 11:**
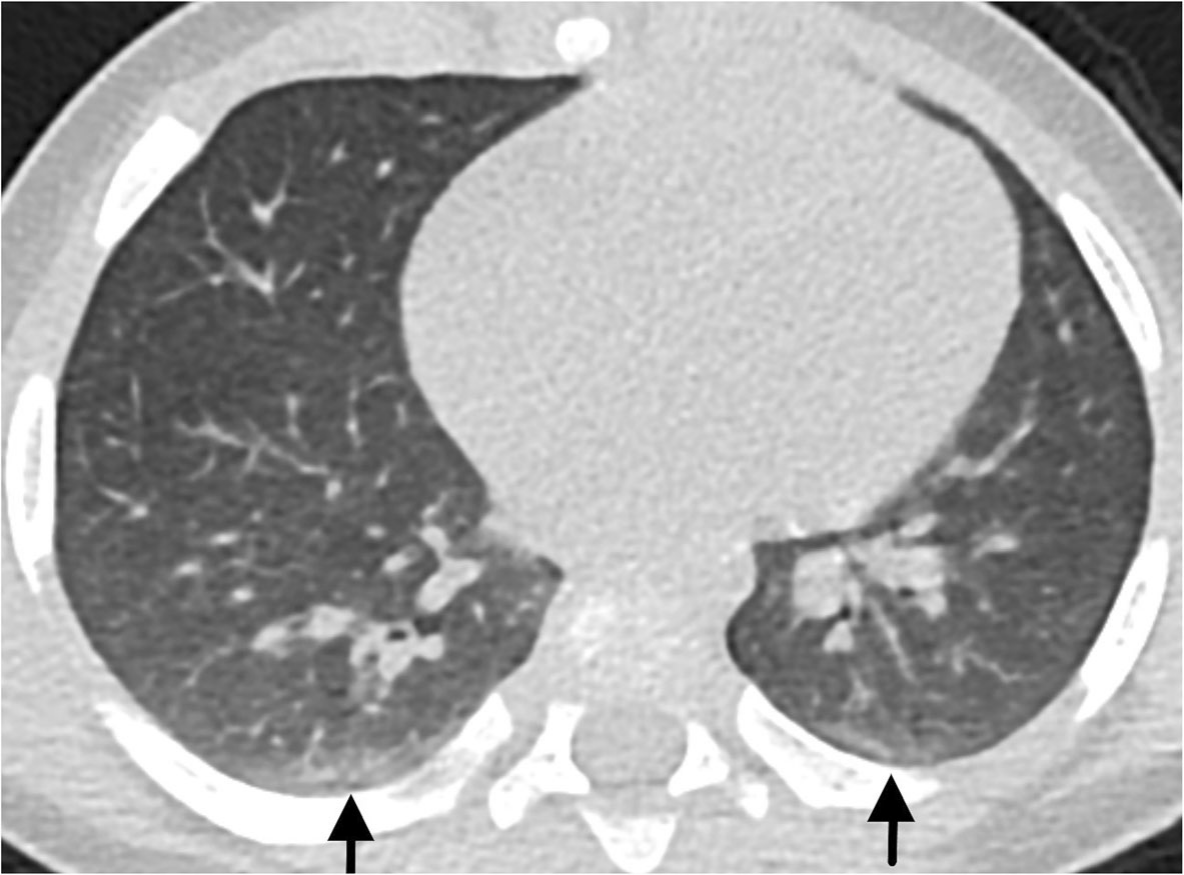
Normal/negative CT findings in a 7-month-old male—linear subpleural opacities in the posterior lungs (black arrows), commonly documented in the dependent position of the lungs of normal patients (due to athelectasic bands caused by hypoventilation/sedation)

Normal CT findings were seen in 7 patients (Fig. 11).

## Discussion

Clinical and radiological features of COVID-19 have been mainly described in adults. Literature states that children show similar but less pronounced chest CT findings comparing to those of adults [9]. In another 20-patient pediatric cohort, Xia et al. [14] reported 20% of normal chest CTs, approximately the same as in this study (29%). In other paper, Wang et al. [5] stated that normal CT findings can be seen particularly in the early stages of the disease. Indeed, 4 of our 7 CTs with normal findings were performed during the first week of symptoms.

Similarly to other studies, typical findings in this cohort included mostly peripheral bilateral GGO opacities. On the other hand, unlike in the cohort from Xia et al. [14], where 50% of the patients showed the halo sign, this typical sign was only seen in 3 of this study’s cases (13%). In the present series, a perilobular consolidation surrounded by GGO was also found, suggesting secondary organizing pneumonia. Despite commonly seen in adults [5], this finding is seldom described in children [1, 9] and not mentioned in the International Consensus [8].

Amongst this study’s indeterminate cases, diffuse GGOs without peripheral or subpleural distribution were the predominant findings and differential diagnosis with other viral pneumonias, atypical bacterial pneumonias and other non-infectious entities was considered [8]. Conversely, one case showed bilateral lower lobe consolidations, with minimum GGO, which may be found in some advanced cases [4] although CT was performed at day 6 of symptoms. Time course of the imaging abnormalities has been evaluated by Pan et al. [4] and Wang et al. [5] in adults. Isolated GGO is typically seen after symptom onset and the authors noticed that opacities tend to evolve not only in extension [4] but also to mixed patterns including crazy paving, consolidation, subsegmental athelectasis, and perilobular abnormalities, the latter suggesting secondary organizing pneumonia [5]. These temporal changes are at least partially responsible for different CT sensitivities along the course of the disease, probably both in adult and in the pediatric population, which may explain some differences between cohorts.

Due to ethical concerns and ionizing radiation exposure risks, serial evaluation of children is not feasible. However, comparing CT findings from different patients in distinct phases of the disease, some authors extrapolated the same temporal pattern from adults to children. For instance, Foust et al. considered that consolidation is part of a later stage of the disease (the “developed phase”), probably evolving from other findings like the halo sign and GGO [8]. Subacute and chronic lung sequalae are yet to be evaluated and are not under the scope of our study.

Besides the halo sign, two other findings have been more commonly reported in children than in the adult population: peribronchial distribution of the opacities and bronchial wall thickening [1]. However, we have only reported peribronchial distribution and bronchial wall thickening in 3 patients (13%). Nevertheless, regarding bronchial thickening, it is not clear if it is only related to SARS-CoV-2 infection or to coinfections (as in one of the cases in this series where a bronchiolitis pattern was seen in a child with concomitant SARS-CoV-2, rhinovirus, and *Mycoplasma pneumoniae* infections). Interestingly, none of the current study patients exhibited a crazy paving pattern (that has also been described as fine mesh with GGO), reported in 20% of the 20-patient study from Xia et al. [14].

Comorbidities constituted a confounding factor while evaluating chest CT in patients with suspicion for COVID-19 pneumonia (including patients with bronchopulmonary dysplasia and asthma). Moreover, coinfection, which is particularly frequent in the COVID-19 pediatric population, further complicated our assessment. Xia and colleagues [14] reported a coinfection rate of 40%, similarly to this study’s data (42%).

Mosaic pattern attenuation was found in 4 of 24 rt-PCR-positive patients (17%); however, it was difficult to correlate this finding with COVID-19 pneumonia. In one of these patients, who also presented bronchial wall thickening on CT, RSV and adenovirus coinfections were identified, suggesting that the mosaic pattern might be related to small airway disease. The other 3 patients had history of prematurity, which might explain the mosaic pattern independently of SARS-CoV-2 infection.

Another confounding factor for diffuse ill-defined GGOs was related to motion artifacts due to lack of collaboration in non-sedated children. Additionally, subpleural opacities may be related to the sedation, potentially leading to passive athelectasic bands that often appear in the dependent lungs.

Some differences were found between this study cohort and other published series, probably reflecting scarce data and lack of consensual guidelines for CT acquisition, namely regarding the appropriate timing.

Besides recognizing typical findings for COVID-19, radiologists should be able to pinpoint those features that favor different or concomitant diagnosis. In this cohort, only 3 laboratory-confirmed cases were considered as atypical, according to the consensus by Foust et al. [8]. With this in mind, we emphasize that normal CT does not exclude COVID-19 and an abnormal CT, even with some suggestive findings, should not be used to diagnose COVID-19 [15].

There are some limitations in this study, namely that it is a retrospective study, with a small number of patients, focusing on patients that performed CT upon confirmed COVID-19 infection. The referral criteria to perform CT were not clearly defined in the early days of this pandemic. Hence, clinical severity was not clearly evaluated in this paper. Also, a significant proportion of our patients had comorbidities and coinfections.

## Conclusions

CT findings in children infected with SARS-CoV-2 can be subtle or absent and may be mistaken for other pathologies. Continuing sharing of case series is crucial for the recognition of the most common features of this still challenging disease.

## Data Availability

The datasets generated and/or analyzed during the current study are not publicly available due to patient confidentiality but are available from the corresponding author on reasonable request. There are no other prior publications or submissions with any overlapping information.

## Abbreviations

ACE2: Angiotensin-converting enzyme II
COVID-19: 2019 coronavirus disease
CT: Computer tomography
CXR: Chest X-ray
GGO: Ground-glass opacity
RSV: Respiratory syncytial virus
rt-PCR: Real-time reverse transcriptase polymerase chain reaction
SARS-CoV-2: Severe acute respiratory syndrome coronavirus 2
WHO: World Health Organization
